# Toward the Growth of Self-Catalyzed ZnO Nanowires Perpendicular to the Surface of Silicon and Glass Substrates, by Pulsed Laser Deposition

**DOI:** 10.3390/ma13194427

**Published:** 2020-10-05

**Authors:** Basma ElZein, Yingbang Yao, Ahmad S. Barham, Elhadj Dogheche, Ghassan E. Jabbour

**Affiliations:** 1 Electrical Engineering Department, College of Engineering, University of Business and Technology (UBT), Jeddah 21361, Saudi Arabia; 2 Institute of Electronics, Microelectronics and Nanotechnology, CNRS and University Lille Nord de France- Avenue Poincaré, CEDEX, 59652 Villeneuve d’Ascq, France; 3 Faculty of Materials and Energy, Guangdong University of Technology, Guangzhou 510006, China; ybyao@gdut.edu.cn; 4 General Subjects Department, College of Engineering, University of Business and Technology (UBT), Jeddah 21361, Saudi Arabia; ahmad.s@ubt.edu.sa; 5 Campus Le Mont Houy, IEMN CNRS, Polytechnic University Hauts de France, CEDEX, 59309 Valenciennes, France; Elhadj.Dogheche@uphf.fr; 6 Canada Research Chair in Engineered Advanced Materials and Devices, Faculty of Engineering, University of Ottawa, Ottawa, ON K1N 6N5, Canada; gjabbour@uottawa.ca

**Keywords:** zinc oxide, seed layer, vertically oriented nanowires, polar nanowires, glass/ITO substrates, pulsed laser deposition

## Abstract

Vertically-oriented zinc oxide (ZnO) nanowires were synthesized on glass and silicon substrates by Pulsed Laser Deposition and without the use of a catalyst. An intermediate c-axis oriented nanotextured ZnO seed layer in the form of nanowall network with honey comb structure allows the growth of high quality, self-forming, and vertically-oriented nanowires at relatively low temperature (<400 °C) and under argon atmosphere at high pressure (>5 Torr). Many parameters were shown to affect the growth of the ZnO nanowires such as gas pressure, substrate–target distance, and laser energy. Growth of a c-axis-crystalline array of nanowires growing vertically from the energetically favorable sites on the seed layer is observed. Nucleation occurs due to the matching lattice structure and the polar nature of the ZnO seed layer. Morphological, structural, and optical properties were investigated. X-ray diffraction (XRD) revealed highly c-axis aligned nanowires along the (002) crystal plane. Room temperature photoluminescence (PL) measurements showed a strong and narrow bandwidth of Ultraviolet (UV) emission, which shifts to lower wavelength with the increase of pressure.

## 1. Introduction

**Figure 1:**
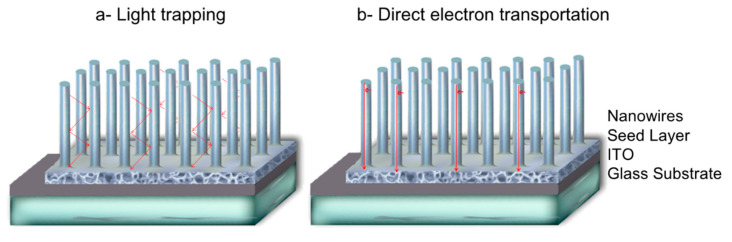
Illustration of (**a**) light trapping in nanowires arrays and (**b**) electron transport in vertical nanowires.

One-dimensional nanometer-sized electrically conducting and semiconducting nanowires (NWs), nanotubes, and nanorods have attracted much attention due to many exciting attributes including a direct path for charge transport and a large surface area for light harvesting. Such characteristics make them excellent candidates for many applications including solid-state lighting and photovoltaics. Freestanding NWs array morphology is favorable to light trapping where the incident light scatters within its open interiors. The scattering improves the efficiency of light absorption by increasing the photon path length. Due to the high electron mobility (reaching tens cm^2^ V^−1^ S^−1^) [1], photo-generated charges are transported quickly to the electrode, especially when the NWs are vertically-oriented with respect to it (Figure 1).

Due to the unique properties of ZnO such as a large direct band gap of 3.37 eV and exciton binding energy of 60 meV [2,3,4,5,6,7,8], it has been employed in numerous applications such as solar cells, light emitting diodes (LED), optical switches, and waveguides, to mention a few. For example, ZnO NWs-based solar cell research has become a hot topic in science and engineering [9,10,11,12,13,14,15,16,17,18,19]. Device architecture having radial, axial, or substrate junction has also been explored [20]. These architectures have been employed in solar cells using homogeneous and heterogeneous NWs.

There are many approaches for the growth of ZnO nanostructures such as catalytic growth via vapor liquid solid (VLS) mechanism [21,22], thermal evaporation [23,24,25], pulsed laser deposition (PLD) [26,27], hydrothermal growth [28,29,30,31], rapid hydrothermal growth [32,33,34], and wet chemical processing [35,36,37], etc. The choice of the growth technique is dictated by the requirements of the application.

PLD has been recognized as a powerful technique in thin film growth. It can produce high quality epitaxial materials as well as amorphous layers at low temperature. It is also used to produce various nanostructures like nanorods [38,39,40,41,42], nanoparticles [41,43,44,45], and nanowalls [46]. A survey [26,47,48,49,50,51,52,53,54,55,56,57] of the synthesis parameters of ZnO NWs by PLD is presented in Table 1 presenting the growth parameters such as the type of seed layer, temperature, pressure, and distance between substrate and target. It is noticed that growth temperature varies between 500 and 900 °C, pressure > 1 Torr, and relatively short target–substrate distance <3 cm.

In this paper, we report the growth of vertically-oriented ZnO NWs on nanotextured seed layer (SL) of ZnO at high background pressure. Unlike what has been reported in the literature, this method requires only the nanotextured ZnO template for nucleation, and does not use any metal catalyst layer. We demonstrate the growth of vertically-oriented ZnO nanorods on both silicon and glass-ITO/ZnO substrates.

## 2. Materials and Methods

**Figure 2:**
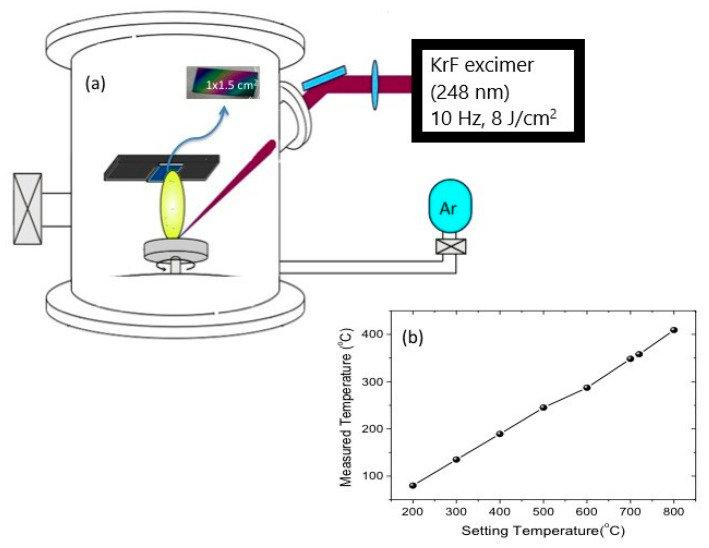
(**a**) PLD setup illustration, (**b**) diagram presenting the measured substrate temperatures in function of the setting temperature.

An ablation source of KrF excimer laser (248 nm) with a repetition rate of 10 Hz and pulse laser energy of 400 mJ/pulse (energy density of ~8 J/cm^2^), and a high purity ZnO target were used (CompexPro 205F, Coherent Inc., Santa Clara, CA, USA). The target was prepared by uniaxial pressing of ZnO commercial powder (99.99% purity from Sigma Aldrich, St. Louis, MO, USA) followed by sintering at 1150 °C for two hours. Prior to deposition; p-type Si (100), and Glass-Indium Tin Oxide (ITO) substrates of 1 × 1.5 cm^2^ were ultrasonically cleaned with a consecutive bath of acetone and isopropanol followed by a drying step using compressed nitrogen. The samples were totally covered by a textured thin layer of ZnO; experimental process is presented elsewhere [58], and then introduced in a high vacuum chamber evacuated to a base pressure of about 10^−6^ Torr. The target-to-substrate distance was maintained at 6.5 cm due to equipment restrictions. Experimental setup details are presented in Figure 2. The substrate was heated to 380 °C (measured temperature) at a rate of 30 °C/min. The temperature was maintained constant during deposition in the presence of argon (>99.99% purity). Structural properties of the as-grown NWs were characterized using Bruker D8 Discover high resolution X-ray diffractometer (XRD, Tokyo, Japan) with CuKα and λ = 1.5406 Å and transmission electron microscopy—FEI—TEM Tecnai (Hillsboro, OR, USA). Morphological properties were examined with FEI Nova Nano SEM 630 (Hillsboro, OR, USA) and Zeiss Ultra 55 (Hillsboro, OR, USA), and photoluminescence properties were studied using Raman Lab with the samples excited using HeCd laser at 325 nm.

## 3. Results

Parametric study was performed to optimize the ZnO NWs growth on various types of substrates with different NWs length, diameter, and density. Morphological, structural and optical properties were investigated.

### 3.1. Morphological Properties—Effect of Seed Layer (SL)

**Figure 3:**
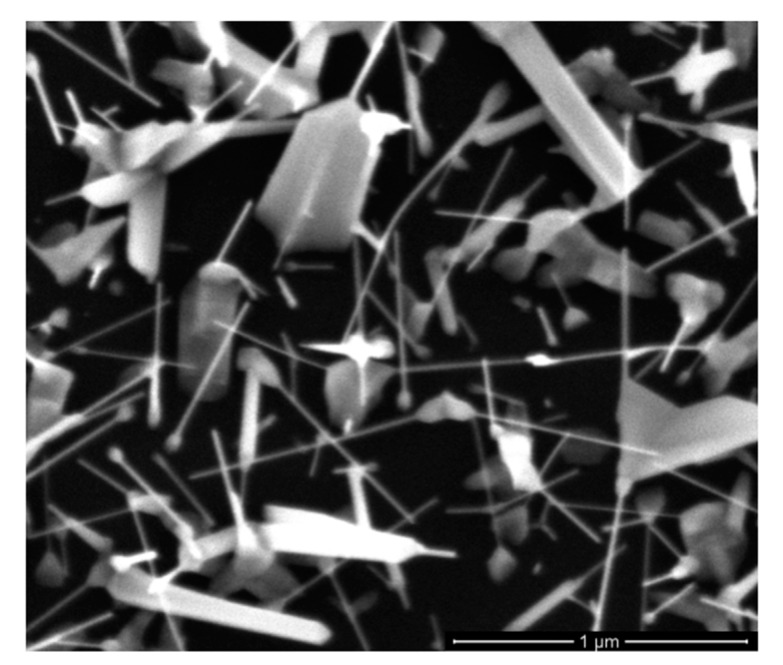
SEM image top view of ZnO nanowires (NWs) grown at 5 Torr in argon environment, T = 380 °C on silicon substrate.

ZnO NWs were grown directly on Si (100) substrates at 5 Torr (argon pressure). Under deposition conditions presented in the experimental section without ZnO SL, the grown ZnO nanostructures showed nail-needle-shape morphology with different dimensions (Figure 3) and random orientation.

A thin layer of ZnO nanowall network with honeycomb structure [58] was deposited as SL on Si (100).

**Figure 4:**
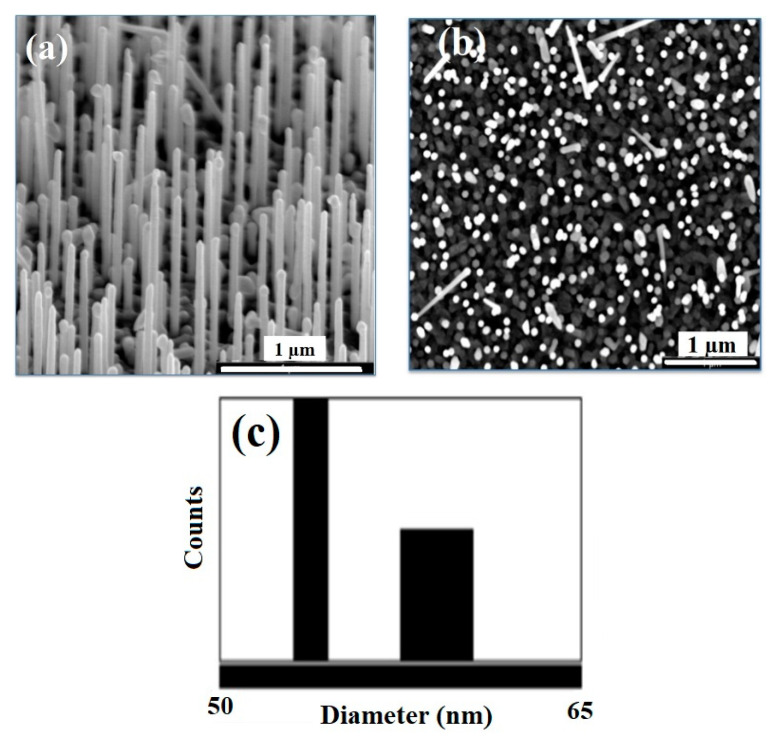
(**a**) 40° tilted view SEM image of perpendicular ZnO NWs arrays grown on ZnO SL/Si (100) substrates at 5 mTorr, (**b**) top view SEM image of the as-grown ZnO NWs, (**c**) corresponding size distribution histogram of the ZnO NWs arrays.

The ZnO textured SL is highly crystalline (c-direction), grown by PLD; growth parameters are presented elsewhere [32]. Under deposition conditions of 5 Torr argon pressure and a deposition time of 30 min, NWs with a perfect vertical orientation were grown on ZnO SL/Si (100) having an average diameter of about 50 ± 4 nm, a length of 1.3 ± 0.12 μm, and spacing of 46 ± 8 nm (Figure 4).

**Figure 5:**
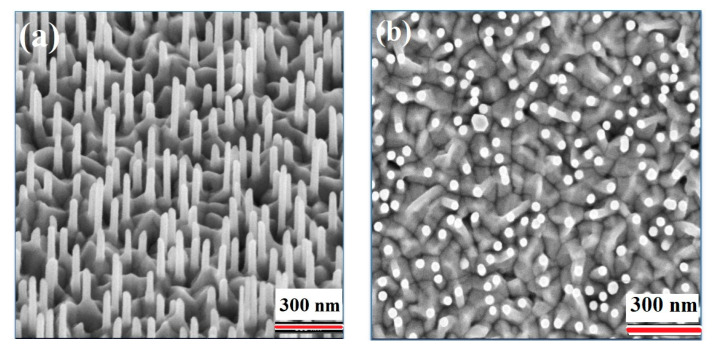

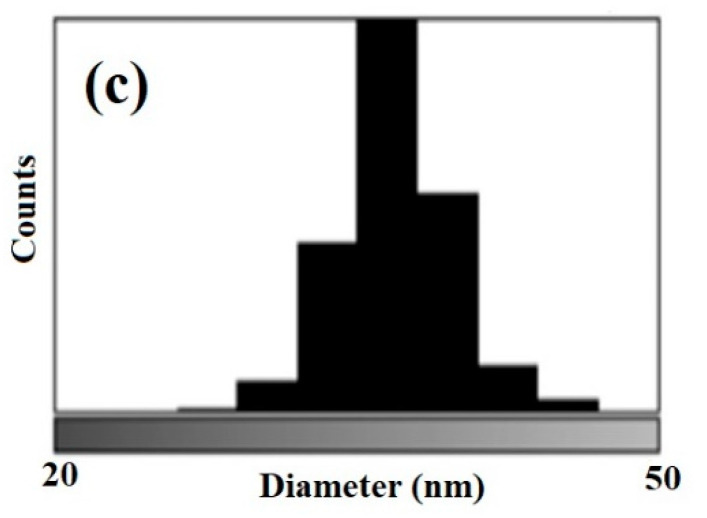
(**a**) 40° tilted view SEM image of perpendicular ZnO NWs arrays grown on ZnO SL/Si (100) substrates at 10 mTorr, (**b**) top view SEM image of the as-grown ZnO NWs, (**c**) corresponding size distribution histogram of the ZnO NWs arrays.

As expected, the geometrical dimensions of the NWs are affected by the deposition conditions. For example, changing the chamber pressure to 10 Torr for 15 min results in 600 ± 30 nm long NWs and having diameter 30 ± 3 nm, with a spacing of 75 ± 5 nm (Figure 5). It is noteworthy that no NWs were obtained at 2.5 Torr background argon gas pressure.

**Figure 6:**
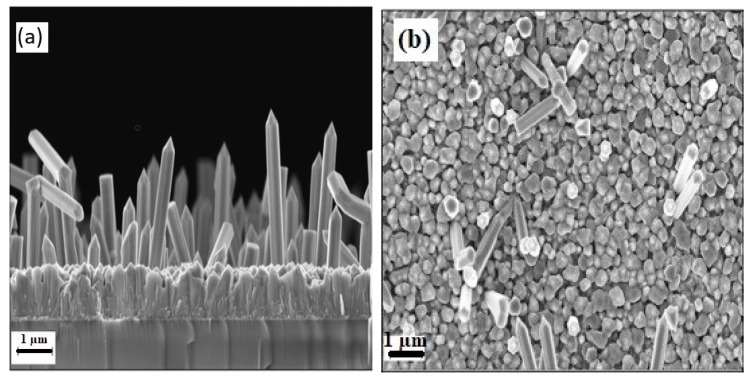
(**a**) Cross-section SEM image of nearly perpendicular ZnO NWs arrays grown on ZnO SL/Glass/ITO substrates at 5 mTorr, (**b**) top view SEM image of the as-grown ZnO NWs.

Under the same deposition, ZnO NWs grew in a pencil-like morphology with 2.6 ± 0.4 μm length and 360 ± 20 nm diameter nearly perpendicular to the surface of glass-ITO/ZnO SL (Figure 6).

### 3.2. Structural Properties

**Figure 7:**
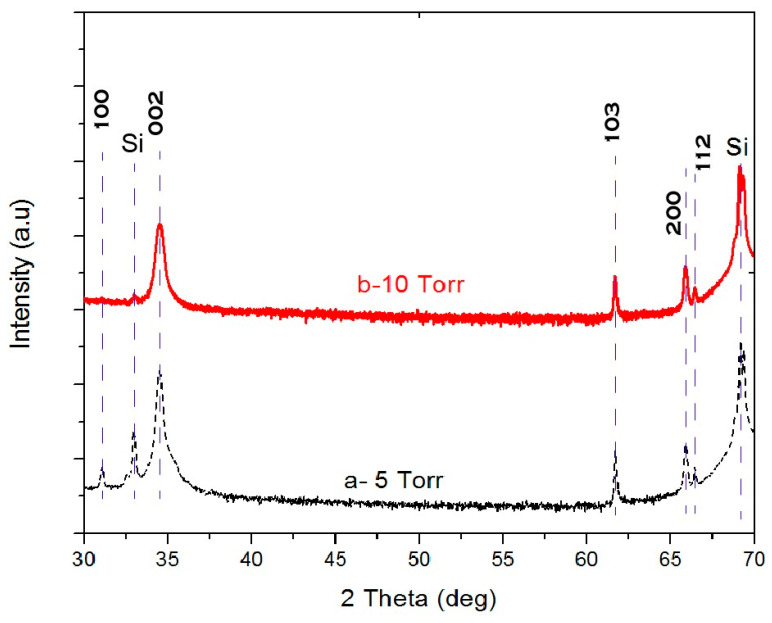
XRD pattern 2θ scan of ZnO NWs grown on ZnO SL at Tsub = 380 °C and argon pressure at (**a**) 5 Torr and (**b**) 10 Torr.

A typical XRD pattern of the ZnO NWs array at 5 and 10 Torr is shown in Figure 7. Only main diffraction lines from (002) and (004) planes can be observed having the highest peak shown at 34.58° and 34.47° for the NWs at 5 and 10 Torr, respectively. It is constructive to note that the NWs array has a c-axis orientation. The other diffraction peaks shown in Figure 7 are due to the silicon substrate and substrate holder. A slight shift can be seen between the two peaks of the (002) plane direction of the ZnO NWs grown at different pressures. This might be caused by the low oxidation of the ZnO NWs due to the background argon environment.

**Figure 8:**
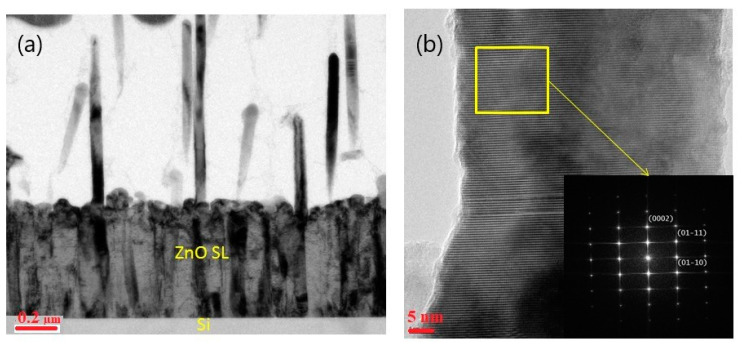
TEM (**a**) & High-Resolution Transmission Electron Microscopy (HRTEM) (**b**) of ZnO NWs grown on Si (100)—ZnO SL at 5 Torr.

The structure of ZnO NWs on ZnO SL was further investigated by TEM. Figure 8 shows a low-resolution image (Figure 8a), HRTEM image (Figure 8b) and selected area electron diffraction (SAED) pattern of a single ZnO NW (Figure 8a). It is clear that the ZnO NWs are relatively straight with a diameter of about 50 ± 4 nm. SAED pattern and HRTEM suggest that the NWs have a single domain wurtzite structure with high crystal quality. The HRTEM image shows a lattice distance of 0.52 nm consistent with the c-axis of wurtzite ZnO crystal. The SAED pattern reveals the exact growth of NWs along the ZnO [2] direction, consistent with the XRD result of Figure 7. The growth of the ZnO NWs is done on the concave tip near the grain boundaries between two ZnO thin grains.

**Figure 9:**
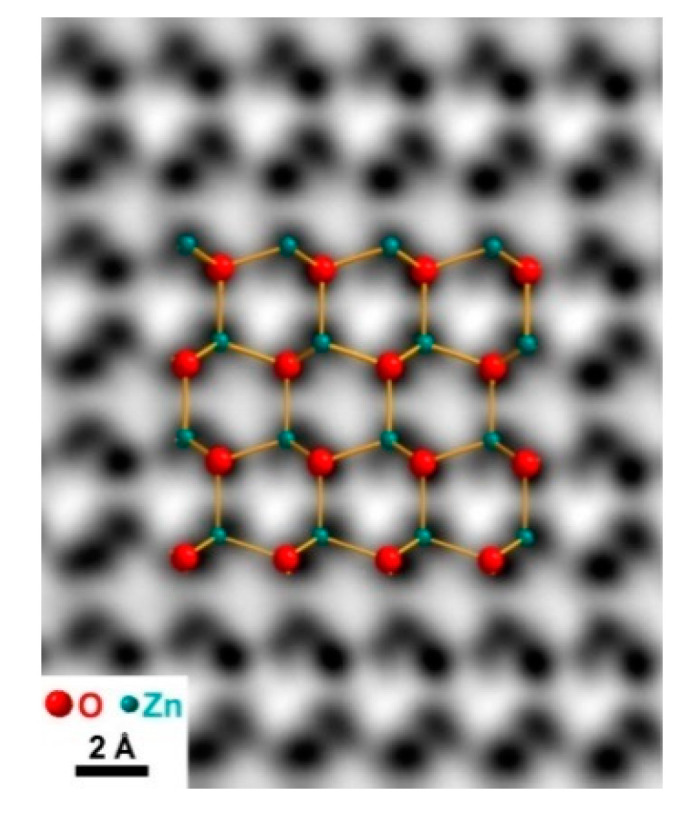
STEM image of ZnO NW with annular bright field (ABF).

When ZnO is viewed along $<11\bar{2}0>$ direction, all the Zn and O atoms are aligned at separate atomic columns, and there is no mixing between Zn and O atoms in the column. This is an ideal case for using STEM (either high-angle annular dark field (HAADF) or annular bright field (ABF)), to study the polarity of ZnO film. However, as Zn and O atoms are only 0.112 nm apart in the $<11\bar{2}0>$ projection, a probe corrector has to be used to achieve such a resolution. Here HAADF is not applicable, as the oxygen light atom cannot be seen due to a weak signal. In this case, ABF is more suitable to study the polarity of the film (Figure 9). Based on the contrast, the position of Zn and O can be accurately identified. As the nanowire is pointing upward, the polarity was identified based on the common definition of polarity of ZnO (the nanowire is Zn terminated [59]).

### 3.3. Optical Properties

**Figure 10:**
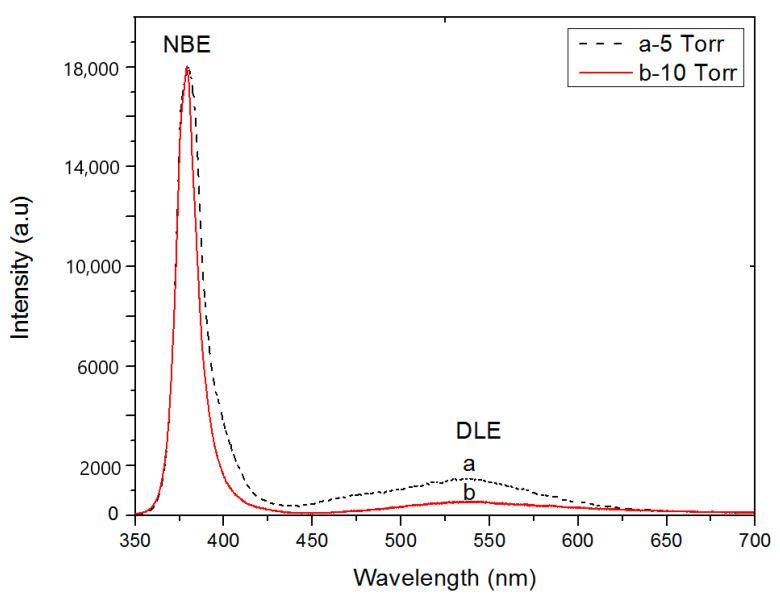
Room-Temperature photoluminescence (RT–PL) spectra of ZnO NWs grown at 5 Torr (**dashed line**) and 10 Torr (**solid line**) argon environment. NBE is the near-band edge emission and the defect level emission is DLE.

Figure 10 depicts photoluminescence (PL) measurements of ZnO NWs grown by PLD at 5 Torr and 10 Torr argon pressure. Different peak positions of the band edge emission in the UV region as well as defect-induced emissions in the visible region can be seen. The PL spectra exhibit normal band-gap emission in the UV region at ca. 379.4 nm (3.268 eV), and 379.2 nm (3.27 eV) for samples grown at 5 Torr and 10 Torr, respectively. A slight shift to lower wavelength is noticed which might be caused by quantum confinement of thinner NWs. The emission in the visible region is namely green (541 nm (2.29 eV), and 539 nm (2.3 eV) for 5 Torr and 10 Torr samples, respectively) and yellow (585 nm (2.12 eV) for both cases). Several types of defects in ZnO can induce emission in the visible region. Table 2 presents the intensity ratio of UV/visible emission. It shows that the UV to green emission and UV to yellow emission for ZnO NWs are higher for working pressure of 10 Torr, indicating fewer defects than the 5 Torr case.

## 4. Discussion

NWs were grown perpendicularly to the surface with a high-density distribution over the entire substrate. The crystal structure of the SL had a considerable effect on the crystallographic orientation of the ZnO NWs.

Many PLD process parameters affect the growth of the ZnO NWs, such as substrate temperature, gas pressure, and the substrate–target distance. The deposition temperature has a critical effect on surface diffusion [26,51,60,61,62]. If it is too low <200 °C, the deposited ZnO will not have enough mobility to reach nucleation sites, and would rather increase the roughness of the surface [63]. An appropriate high temperature would allow the deposited species to migrate to energetically favorable sites where the nucleation energy barrier is lower. This is due to the higher sticking coefficient of ZnO on the nuclei sites. These are likely to be the vicinity of grain boundaries as was demonstrated by TEM analysis. It is worth mentioning that the temperature used in our work (400 °C) is less than in the previously published literature, as well as the target–substrate distance (6.5 cm) being larger [64]. The lower substrate temperature was found to be sufficient for activating surface diffusion. In this research, the pressure used for the growth of ZnO nanowires was 5 Torr–10 Torr, recommended by S. Lemlikchi et al. [65] and R.S.Ajimsha et al. [66]. The average diameter of the NWs grown at 10 Torr is less than that of the 5 Torr. The increase of pressure from 5 Torr to 10 Torr caused the NWs spacing to increase from 46 ± 8 nm to 75 ± 5 nm, respectively, as the argon gas pressure influences both the deposition rate and the energy of ejected species. When the deposition is processed under high pressure, the ablated species undergo a large number of collisions with background gas molecules (argon atoms), which reduces the energy of the particles arriving at the substrate–Seed Layer (SL) and decreases the size of the ablated plume [67]. That is why it is recommended to reduce the distance between the target and substrate while working at higher pressure in order to maintain the optimum energy of the ablated species. The decrease of the kinetic energy is likely to be the reason why thinner NWs with lower density were obtained over the surface of the substrate at 10 Torr. Furthermore, ZnO nanowires were successfully grown on glass-ITO-ZnO SL substrate at 5 Torr. The thickness of the glass substrate also affects the morphology of NWs, due to heat transfer phenomenon.

On the other hand, for the metal oxide such as ZnO, gold (Au) or silver (Ag) catalysts are not needed if Zn can be decomposed of ZnO during the growth of NWs. Having a high melting point, ZnO might have been decomposed and created a self-catalytic Zn nano-dot from the vapor liquid solid process (VLS). ZnO NWs can be grown just above the melting point of Zn. The morphology, density, and uniformity of the NWs depend on the surface and surface migration energies of the substrate.

**Figure 11:**
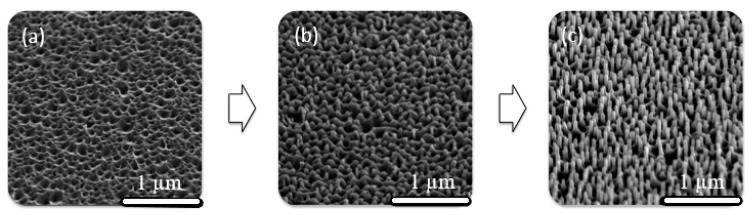
SEM images revealing the growth process of ZnO NWs on ZnO SL by PLD. Ablated ZnO species are adsorbed on the seed layer (SL) nanostructured surface (**a**). ZnO species migrate to the nucleation points that are energetically favorable sites for growth of ZnO NWs (**b**), followed by continuous growth (**c**).

In order to understand the growth process, the deposition of ZnO NWs on ZnO SL was performed using PLD at different deposition durations. Figure 11 reveals the schematic illustration of growth of ZnO NWs grown by PLD on Si substrates having a ZnO SL. It is suggested that growth rate of ZnO along the normal direction is higher than the rate at the different index planes ($V\left(0001\right)>V\left(10\bar{1}0\right)>V\left(10\bar{1}\bar{1}\;\right)>V\left(10\bar{1}1\right)>V\;\left(000\bar{1}\right)$) [68]. The presence of ZnO SL can efficiently lower the nucleation energy barrier leading to nucleation of ZnO NWs. Moreover, the continuous supply of ZnO assists the growth of NWs in a favorable direction [2]. The NW’s length increases with growth time, and the density of the NWs varies with the nucleation sites on the surface of the SL and the argon pressure in the PLD chamber.

## 5. Conclusions

In summary, we have demonstrated that vertically-oriented ZnO NWs could be grown on Si and glass/ITO substrates with an intermediate nanostructured ZnO SL by pulsed laser deposition at relatively low temperature under high argon pressure. Since no intentional metal catalyst was introduced, the incorporation of a textured ZnO SL was a key for the growth of the desired NWs. The grain boundaries of the used ZnO SL were found to be the most favorable nucleation sites. The as-synthesized NWs, found to present Zn polarity, were c-axis oriented in agreement with the SL crystallinity. This is a promising substrate-independent growth method for fabricating aligned NWs on a large scale to be applied in photovoltaic, light emitting diodes, electronic devices for improved light trapping, and other electronic devices.

## 6. Patents

The patent number “US 2015/0280017 A1” resulted from the work reported in this manuscript.

## Figures and Tables

**Table 1:** Survey of ZnO nanowires and nanorods by pulsed laser deposition (PLD).

Substrate	Temp (°C)	Pressure (Torr)	Distance Between Target and Substrate (cm)	Diameter (nm)	Length (µm)	Ref
Sapphire (0001)	600–700	1–5	2	300	6	[47]
Si (100)	450–500	5	2.5	120–200	12	[48]
SiO_2_/Si/Au	900	400	-	20	10	[49]
Sapphire (0001)	600	5	2	300	6	[50]
Si (100)	600–850	4.8–6.3	2.5	20–50	0.5–2	[51]
a-Sapphire c-Sapphire	1000	260	1.5	200	0.5–3	[52]
c-Sapphire ZnO SL	500–800	0.15–0.50	2.5	50–90	Few µm	[26]
Sapphire (0001)	-	260	1.2–2.5	130–200	1.5–4	[53]
c-Sapphire	600	0.1–0.2	5	150–200	0.9	[54]
Sapphire	650	10^−2^	5	-	-	[55]
a-Sapphire c-Sapphire + Au	870–950	18–150	0.5–3.5	150	1.5–20	[56]
n-doped 400 µm Si (111)	500–600	0.225	3	-	-	[57]
Si(100) + ZnO Seed Layer	380	5	6.5	50 ± 4	1.3 ± 0.12	This work
Si (100) + ZnO Seed Layer	380	10	6.5	30 ± 3	0.6 ± 0.03	This work
Glass/ITO + ZnO Seed Layer	380	5	6.5	360 ± 20	2.6 ± 0.4	This work

**Table 2:** Intensity ratio UV/visible of ZnO NWs deposited at 5 Torr and 10 Torr, respectively.

Sample	UV/Green	UV/Yellow
NWs at 5 Torr	11.868	22
NWs at 10 Torr	30.6	45.4
